# Care-seeking behaviors for maternal and newborn illnesses among self-help group households in Uttar Pradesh, India

**DOI:** 10.1186/s41043-017-0121-1

**Published:** 2017-12-21

**Authors:** Kumudha Aruldas, Aastha Kant, P. S. Mohanan

**Affiliations:** 1 Population Council, New Delhi, India; 2 Rajiv Gandhi Mahila Vikas Pariyojana, Raebareli, Uttar Pradesh India

**Keywords:** Care-seeking, Maternal illness, Newborn illness, Social platform, Self-help group, Uttar Pradesh, India

## Abstract

**Background:**

India has made large strides in reducing maternal mortality ratio and neonatal mortality rate, yet care-seeking behavior for appropriate care is still a challenge. We conducted a qualitative study to understand the process of recognition and care-seeking for maternal and newborn illnesses in rural India where a health intervention through women’s self-help groups (SHG) to improve maternal and newborn health behaviors is implemented by a non-governmental organization, the Rajiv Gandhi Mahila Vikas Pariyojana. The study aimed to understand the process of recognition and care-seeking for maternal and newborn illnesses from SHG and non-SHG households in the intervention area.

**Methods:**

Thirty-two illness narratives, 16 of maternal deaths and illness and 16 of newborn illnesses and deaths, were conducted. Women, their family members, and other caretakers who were present during the event of illness or death were included in the interviews. About 14 key informants, mainly frontline health workers (FLWs), were also interviewed. The interviews were conducted by two Population Council staff using a pre-tested guideline in Hindi.

**Results:**

Our findings suggest that perceptions of causes of illness as “supernatural” or “medical” and the timing of onset of illness influence the pathway of care-seeking. Deep-rooted cultural beliefs and rituals guided care-seeking behavior and restricted new mothers and newborns’ mobility for care-seeking. Though families described experience of postpartum hemorrhage as severe, they often considered it as “normal.” When the onset of illness was during pregnancy, care was sought from health facilities. As the step of care for maternal illness, SHG households went to government facilities, and non-SHG households took home-based care. Home-based care was the first step of care for newborn illnesses for both SHG and non-SHG households; however, SHG households were prompt in seeking care outside of home, and non-SHG households delayed seeking care until symptoms were perceived to be severe.

**Conclusion:**

Our findings indicate that care-seeking behavior for maternal and newborn morbidities could be improved by interventions through social platforms such as SHGs.

## Background

Globally, the maternal mortality ratio (MMR) declined from 385 to 216 per 100,000 births between 1990 and 2015 [1]. In a similar timeframe, India achieved significant strides in reduction of its MMR––going from 556 per 100,000 live births in 1990 to 174 per 100,000 live births in 2015 [1]. While in 1990, almost 150,000 women died in India due to maternal complications, making up 27% of global maternal deaths [2]; by 2015, there were 45,000 deaths in India, contributing to 15% of global maternal deaths [1]. The maternal mortality ratio declined most rapidly during the period 2004 to 2006, coinciding with the beginning of the National Rural Health Mission and other government programs such as *Janani Surakshya Yojana* of the Government of India (GoI) that encouraged institutional deliveries [2]. The GoI has extended the program to provide services to all sick newborns by including ambulance services, drugs, and diagnostics for newborns under the *Janani Shishu Suraksha Karyakram* program [2]. The neonatal mortality rate stands at 28 per 1000 live births, comprising 57% of all deaths in childhood up to 5 years of age in India [2].

Studies have shown that in South Asia, care-seeking is low for newborn illness, especially in terms of care sought from health facilities and medically trained providers [3]. Care-seeking for maternal illness in settings with many home births, from anthropologic literature, also showed that lack of awareness of danger signs of maternal illness influences the decision-making process to seek care, especially in the post-partum period [4].

Understanding the process of illness recognition and care-seeking of families in large scale maternal and newborn intervention areas is the key to strengthening interventions and improving maternal and newborn health outcomes. Furthermore, the literature shows a 4–30% improvement in seeking care from medically trained providers, as a result of community-based interventions, for essential newborn care, birth preparedness, and community mobilization through women’s participatory groups [3, 5–9]. Based on the available literature, a conceptual model was developed by Moran et al., for maternal and newborn illness recognition and care-seeking [10]. This model included enabling factors and barriers for illness recognition and care-seeking at the individual, family, community, and health system level. Based on a conceptual model, this study was designed within the context of an ongoing self-help group (SHG) program. We thus studied care-seeking and illness recognition patterns in the intervention area in the state of Uttar Pradesh (UP) in India among families wherein women were participating and not participating in SHGs for economic and health empowerment activities. The study objectives were to understand (i) the processes of recognition and care-seeking for maternal and newborn illnesses, (ii) the sequences of actions for care-seeking by families experiencing maternal and newborn illnesses, and (iii) the way health interventions using SHG platforms influence care-seeking for mothers and newborns’ illnesses.

### Intervention

The SHG initiative of *Rajiv Gandhi Mahila Vikas Pariyojana* (RGMVP) started with an aim to build capacity of women in microfinance to bring them out from the cycle of poverty, build their social capital, and introduce an empowerment agenda for them to demand their government entitlements. The SHG consists of 10–12 women, including pregnant and lactating women, among others. One member from each SHG was trained on maternal and newborn health over a period of 6 days to work as a peer health educator called *Swasthya Sakhi*. A flip book, *Facts for Life*, was used to create awareness and increase knowledge among SHGs. The health intervention was to implement a health behavior change management model addressing maternal and newborn care among SHGs. The maternal health component includes identification of danger signs and referral, birth preparedness and complication readiness plan, family planning, and promotion and facilitation of antenatal care and postnatal care. The newborn health component includes skin-to-skin care, early and exclusive breastfeeding, cord care, immunization, and identification of newborn danger signs. The peer health educator discussed health issues among their SHGs, once a month for about an hour. The peer educators established links with the public frontline health workers (FLWs) called Accredited Social Health Activists (ASHAs) to facilitate access to preventive and curative services. In the year 2016, RGMVP recorded more than 1.4 million households in 42 districts with approximately 65,000 SHGs trained on reproductive, maternal, and neonatal health [11].

## Methods

### Data collection

Maternal and newborn deaths are recorded in the project management information system (MIS) of RGMVP. Block-wise MIS data of newborn deaths from two districts of Uttar Pradesh, Raebareli and Amethi, were accessed, and administrative blocks with the highest number of newborn deaths were selected. Block-level staff of RGMVP were then contacted to obtain village-wise list of newborn deaths and maternal deaths. From the list of villages with the highest number of newborn deaths or any maternal deaths, the FLWs were contacted to confirm the deaths. Since there were no data on morbidity available in MIS, the list of all women who had delivered within the past 6 months from the date of the visit was taken from FLWs’ pregnant women registers. Home visits were made to all houses where there was a delivery to enquire if they had faced PPH or neonatal illness. The inclusion criteria of the study were applied to all the PPH and newborn illnesses identified. The inclusion criteria were that the event (illness or death) should have occurred within 6 months from the date of interview. For the selection of maternal death cases, the criteria were women 15–49 years of age who died during pregnancy or within 42 days of childbirth from an illness. For PPH cases, a woman should have had a live birth and perceived that her bleeding from the birth canal post childbirth to be heavy or severe. For newborn illness and death cases, the event should have occurred within 28 days of birth and the onset of illness should have been at home. All the eligible cases from SHG and non-SHG households from the selected villages were interviewed. A total of 32 illness narratives were collected from 25 villages.

Of the 32 illness narratives, ten were post-partum hemorrhage (PPH), six were maternal deaths, ten were newborn illnesses, and six were newborn deaths. In addition, 14 key informants, mainly FLWs, were also interviewed. Interviews were conducted in Hindi during the months of March–April 2015 by two Population Council staff with two female investigators as note takers. Two male investigators interviewed key informants and husbands.

For the illness narratives, data were collected from women, as well as family members, who reported maternal deaths, postpartum hemorrhage (PPH), or newborn illnesses or deaths. The community members including neighbors and ASHAs, who were present during illness, were included in the illness narratives. Husbands and other male members who refused to participate in the group interview were interviewed separately with their consent.

### Data analysis

Data analysis consisted of data reduction, data display, and conclusion drawing and verification, following the Miles and Huberman approach [12]. Data reduction was the first step, whereby data were sorted and organized using Atlas.ti version 6.2. Hindi transcripts were loaded into Atlas.ti and coded. Data display was the second stage, whereby all similarities and differences among cases were recognized as data patterns by organizing their codes in a matrix that allowed data to be displayed for within-case analysis and cross-case analysis. This was followed by defining domains that showed patterns more clearly and allowed for differentiation between maternal illnesses and deaths, and newborn illnesses and deaths. The third step gave meaning to the data through explanations and possible configurations to arrive at results.

### Ethics approval and consent to participate

Written or verbal consent was obtained prior to all interviews. The Institutional Review Board of the Population Council in New York approved this study after a complete review on February 19, 2015.

### Profile of the participants

Women who participated in the study belonged to extended families were Hindu by religion and belonged to scheduled caste. The age of women ranged from 20 to 37 years, and they had mean of 2.9 children.

## Results

### Factors influencing recognition of illness and severity

The analyses of the narratives showed that women and their families easily recognized the symptoms of maternal and newborn illnesses based on the past experience of self or others. They were also able to assess the severity of symptoms by the frequency and amount of the symptom, as well as change from normal behavior, such as frequent episodes of vomiting milk and change in the tone of cry of the newborn. The care-seeking behaviors were dependent on the following factors:

#### Perceptions of causes of illness

We classified the causes of maternal and newborn illness as perceived by older women in the family as supernatural or medical. The perceived causes of maternal illnesses were largely “medical” in nature, while for newborn illness, the perceived causes were both medical and supernatural (Table 1).

**Table 1:** Perceived causes of postpartum hemorrhage and maternal death

Perceived as medical cause of PPH	Perceived as supernatural cause of PPH
○ Bad blood accumulated during pregnancy comes out after delivery ○ Lifting heavy objects such as cot opens up a weak uterus causing bleeding ○ When placenta ruptures, there is bleeding ○ Accidental falls during pregnancy causes bleeding	○ Effect of evil spirit––fear because of hearing unnatural sounds causes bleeding ○ Cat crossing new mother’s path is a bad omen and causes bleeding
Perceived as medical cause of death	Perceived as supernatural cause of death
○ Poisoning and swelling of a woman’s body due to a dead fetus ○ Fits due to less blood in the body ○ Continuous bleeding after delivery causes weakness and death ○ High blood pressure, swelling of the body, and breathlessness ○ Bleeding due to ruptured uterus ○ Mental tension (due to marital discord) lead to shock, cold body, and death	○ Effect of evil spirit in the form “bad” air

PPH was also sometimes perceived to be related to supernatural causes, specifically the effect of an evil spirit (*hawa bayar*), if post-partum bleeding did not stop after taking medication. The amount of bleeding, in comparison with previous deliveries and experiences of other women in a family, aided in the recognition of PPH. Women described severity of bleeding in terms of amount and duration:

*“If the blood was collected in a box, it would have been at least two kilos.”* (woman with six children)

*“On the first day of delivery, almost one liter of black colored blood must have flowed. Black-black blood clots were coming out. Blood clots must have easily weighed a kilogram.”* (woman with six children)

*“On the ninth day of delivery after I took bath for the first time, I started bleeding… My sister-in-law saw the blood on the floor and told me that I am bleeding a lot. She also told my mother-in-law that I have bled a lot and to get medicines.”* (woman with one child)

*We returned from the hospital and then I went with my sister-in-law to a field to defecate…just as I sat, I heard some sound…after I came back home I started bleeding profusely. We called FLW in the morning…she gave medicines…when medicines had no effect on me then we all knew that evil eye had cast a spell on me…my mother went to a traditional healer…he gave blessed cloves to eat…I recovered.* (woman with two children)

On the other hand, there is a tendency in the community to normalize post-partum bleeding. It is commonly believed that women can bleed for about a month after giving birth. Subsequently, normalizing symptoms of bleeding becomes a reason for not seeking care for perceived excessive bleeding. Below are some of the excerpts of women:

*“I bled for almost two months, but my mother kept saying that this happens to women after delivery…she is more experienced…so I did not take any medicine to stop bleeding.”* (woman with two children)

*“I was bleeding a lot, I was scared…my mother-in-law told me to wait for a couple of days...then after two days I told her again…she said to wait…it happens sometimes.”* (woman with one child)

The family members who experienced a maternal death in their household commented on the severity of symptoms that facilitated them to perceive causes of illness as:

*Despite being so ill…she would start laughing seeing her mother-in-law and she would start crying seeing the doctor…there had to be some reason...something was wrong with her…maybe an effect of supernatural spirit* (aunt of the deceased woman)

*Her stomach was cold and did not feel as though the baby was moving, she was throwing her hands and legs. Her eyes had started to roll up, it seemed as if she was about to die* (sister-in-law of the deceased woman)

Based on the symptoms of newborn illness, as illustrated in Table 2, the older and experienced women in the family identified the cause of illness either as medical or supernatural. The perceptions of causes of illness influenced the choice to seek care from healthcare providers for medical causes or traditional healers for supernatural causes.

**Table 2:** Classification of newborn symptoms as per perceived causes of illness

Symptoms of medical cause	Symptoms of supernatural cause
○ Skin eruptions ○ Bloated stomach ○ Loose motions ○ Cold ○ Too weak ○ Jaundice ○ Pneumonia ○ Umbilical bleeding	○ Changes in skin color to blue, black, and yellow ○ Stiffening of body ○ Non-stop crying ○ Getting jitters ○ Not taking breastfeed or drinking milk ○ Crying like a bleating goat ○ Vomiting milk

### Decision-making and care-seeking patterns

Families reported that elder women made decisions about care during pregnancy and childbirth, based on their experiential knowledge of childbearing. Experienced women of the household reinforce the practice of restricting new mothers’ movement within the house as new mothers are considered ritually impure after delivery. Women decide if care should be given at home or sought from outside of the home. If decided to seek care from outside, men take decision on place of care and also arrange for transport and finances to access care. As quoted below, key informant interviews also revealed that the decision-making process tends to rest with the experienced women and older men of the household.

*“It is usually the mother or mother-in-law who identifies the complications among women and children and make decisions on care-seeking … if not, the grandmothers decide.”* (frontline health worker)

Decisions regarding the causes of symptoms and care-seeking actions are also influenced by neighbors and other community influencers. Decisions of care depend on the perceptions of the causes of symptoms. If family members attribute a supernatural meaning to the cause of symptoms, then care is usually within the household or sought within the community, whereas, if the cause of a symptom is considered to be medical, then care is sought from health facilities. The men decide on the type of facility to seek care from, public or private.

With respect to decision-making about the type of facility, some families preferred private clinics due to their close proximity and accessibility at times of emergency. Previous good experience from private clinics, either their own or others’, also influenced decisions to seek care from private clinics. For instance, neighbors told a family which hospital to go to, as they had gone to the same place when their child had similar illness. Families reported that even in private clinics and hospitals, there are child specialists, and they have medicines for all ailments. Public health facilities are the first point of contact when a particular hospital or its doctors are known to the family or neighbors. The child is taken to a higher-level facility if a doctor refers them.

### Timing of onset of illness

**Fig. 1:**
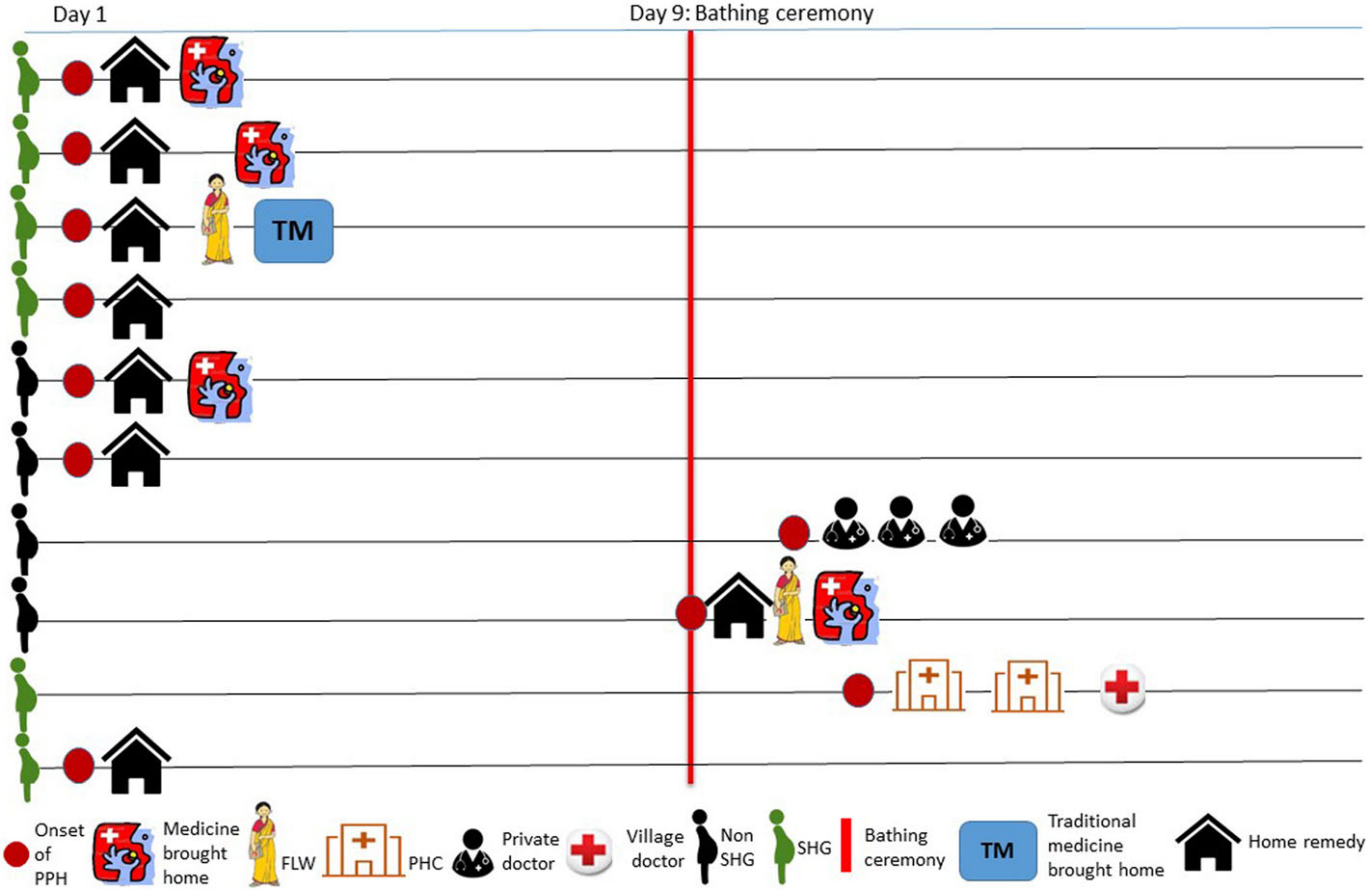
Timing of onset of PPH and care-seeking pattern

Most women interviewed had delivered at a health facility and experienced PPH on the same day after getting discharged from the facility and reaching home, even though they had delivered in a health facility. Further, the decision of accessing care from a facility is also influenced by the timing of the onset of the illness. For instance, if a woman experiences excessive post-partum bleeding before her social ceremony on the ninth day of childbirth, which marks the end of the “polluting” period, then older women ensure that the treatment is brought home. However, after the ceremony, families have taken women to seek care from the facilities. Figure 1 illustrates that the first step of care-seeking is restricted to the household, including bringing medicines home or taking home remedies to expel blood. Only after the ninth day do women seek care from outside their households, at a public or a private health facility. The ninth day marks the bathing ceremony (*nikasan*), a ritual that ends the “pollution” related to childbirth, after which women can leave the house to seek care.

Of the six maternal deaths studied, five of them experienced illness during pregnancy and one after delivery. Illness during pregnancy was perceived as alarming, and consequently, the families sought care at a private or public facility. Families moved between public and private facilities for care, as well as across public and private health facilities. Among SHG households, the first step of care was from government health facilities. Non-SHG households adopted home-based care as their first step of care. Home-based care included either calling traditional birth attendants or undertaking home remedies. However, for the second and subsequent steps of care, non-SHG households also sought care from health facilities.

The timing of onset of illness also influenced the care-seeking behavior for newborn illnesses. Both SHG and non-SHG households chose home-based care as the first step for newborns less than 9 days old. This pattern could be explained in the light of community’s beliefs regarding vulnerability of newborns to evil spirits, especially in the first 8 days when both mother and child are confined to a room until the bathing ceremony (*nikasan)* occurs.

*“Until the ninth day after delivery when nikasan is performed, they (mother and newborn) are vulnerable to ‘evil air’, which may harm them…. so they are kept in one room. We call this period saur. The room should not have windows and have only one door…it should be closed from all four sides so that evil air cannot enter the room….no one can come in or go out…iron objects are kept in the room to ward off evil spirit. Otherwise it is said that some insect [evil] may come and harm the child.”* (sister-in-law)

For newborns less than 9 days old, the families preferred household-level care as the first step of care for newborn illnesses, such as bringing medicines like mentholated topical ointment or calling traditional healers or FLW to visit the home. For subsequent steps of care, families took their newborns to private or public facilities. Some families took as many as six steps of care for their ill newborn. This pattern was seen across SHG and non-SHG households.

**Fig. 2:**
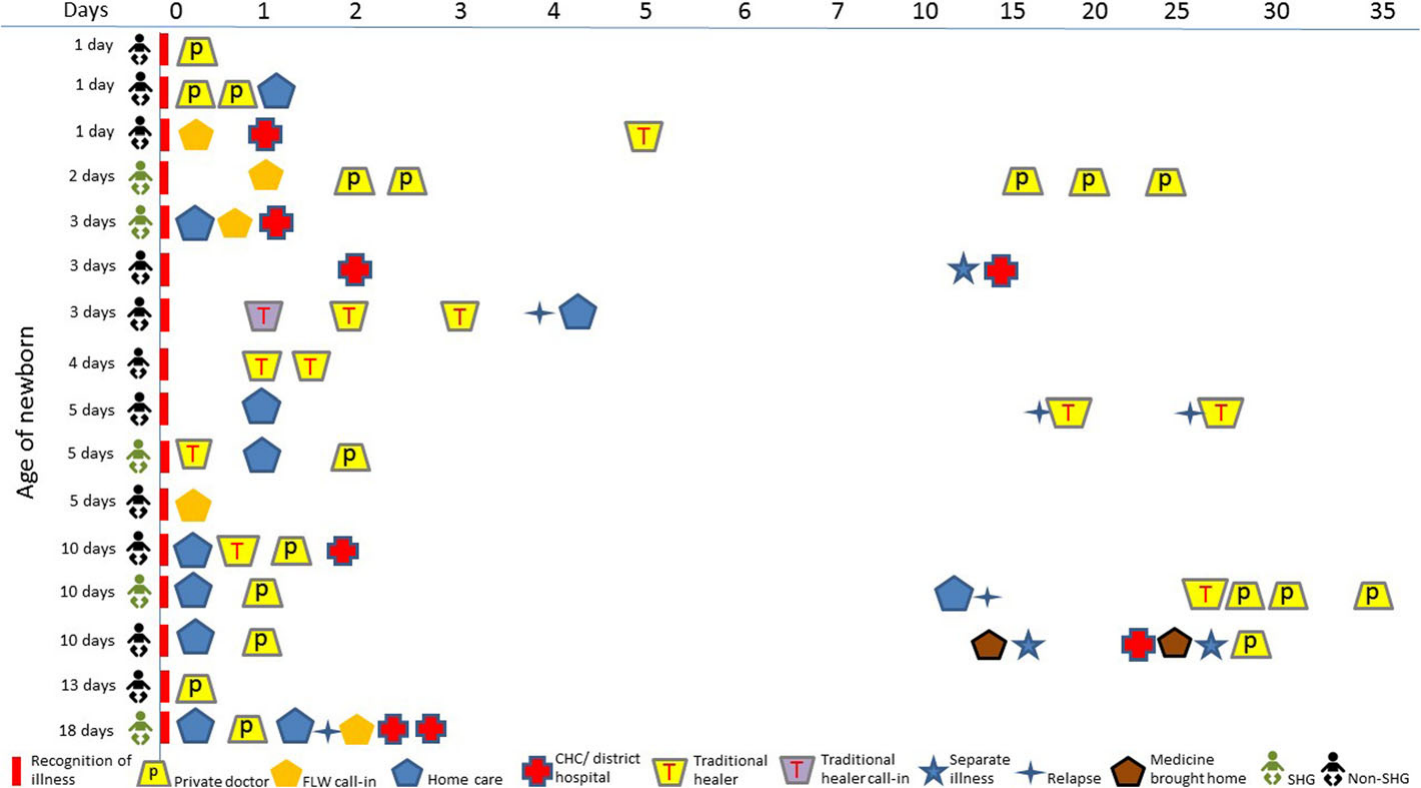
Care-seeking patterns for newborn illness (illness and death cases)

Figure 2 represents the timing of care-seeking for all 16 newborn illness and death cases. Although patterns of care-seeking for the early neonatal period were similar among SHG and non-SHG households, the SHG households sought care from providers on the same day as the onset of illness and the non-SHG households delayed care-seeking by at least a day from the time of onset.

The care-seeking pattern for newborn cases who fell ill after 8 days of birth also reveals that household level care was the first step of care that included home remedies; however, there were no delays in seeking care outside the home for illnesses if the onset of the illness occurred on the ninth day after birth. If the illness occurred at night in winter, the families waited until the morning as they thought they would not be able to get a closed vehicle at night that could protect them from cold.

### Facilitators for care-seeking

#### Accessibility to private health care providers

Services from private healthcare providers were common for postpartum bleeding and newborns who fell ill on the ninth day or later. The private doctors in the neighborhood are considered good as they are popular, and care from a higher level facility is sought only when there is no improvement. Availability of medical shops in the vicinity also facilitated care, as families could buy medicines directly from medical shops.

#### Quality of care

The non-responsive behavior of public hospital doctors was described as another reason to prefer private facilities despite doctors’ referral to a public hospital. Family members felt that doctors did not tell them enough about women’s conditions. A husband referring to his experience of taking his wife to a public health facility said,

*“The nurses in government facilities do checkup as their duty, but do not take care of patients. They will come when they feel like coming, not when we ask them to come and see our patient. In private [hospital], they keep checking the patient again and again. In a private hospital, a nurse will immediately check your patient when we ask her to do.”*

A woman referring to her sister-in-law, who died of PPH, mentioned,

*“We went everywhere [primary health center, district hospital, private hospital, tertiary hospital] but doctors could not control her bleeding…they did not tell anything about why she was bleeding.”*

Health providers’ non-responsive behavior was also seen as poor quality of care for newborn illness and served as a hindrance to care-seeking.

#### Presence of frontline health workers

Within communities, FLWs facilitated care-seeking during pregnancy and labor. As one of the FLWs said,

*“There is also an ambulance service, which can be arranged, that is why people come to me; they feel I can make arrangements.”*

FLWs also decide which level of public health facility to go to. If they feel that the doctor may not be available at the primary health center (PHC), then they take the women to community health centers (CHC), where doctors are always available.

#### Previous experiences of treating illness at home

Previous experiences by the mothers or others of successfully treating newborn illness at home influenced home-based care at the first step of care when similar symptoms were experienced again. For instance, previous experience of treating cold by applying mentholated ointments or umbilical pus by applying penicillin was the reason for opting the similar care at home when the newborns experienced similar symptoms. In addition, community women known for giving massage to the newborns (*nain*) also suggested home-based care on the basis of their experiential knowledge of treating newborn illnesses. As one woman said,

*“*Nain *was called for massaging the child…she recognized the symptoms of cold…asked us not to use* bukwa *[paste of mustard seeds], and instead suggested to use mustard oil.”* (woman with one child)

In another instance, a woman mentioned that her child was bleeding from the navel and she applied penicillin ointment because her neighbor had recommended it since her child also had similar problem which stopped after applying penicillin. Thus, the first step of care was at the household level by buying penicillin from a medical store for application.

### Barriers in care-seeking

The study also revealed the following cultural, financial, and other barriers to care-seeking:

#### Cultural barriers

Cultural beliefs and practices around childbirth restrict women’s mobility to the home and hinders leaving the home to access treatment. Women are expected to perform the purifying bathing ceremony on the ninth day on an empty stomach. When one woman experienced excessive bleeding on the ninth day after childbirth, the family did not give her the medicine until the ceremony was over:

*“She started bleeding profusely just before her ritual bath…we got the medicine but did not give her immediately because the bathing ritual was to be observed on empty stomach.”* (mother-in-law)

Another cultural barrier to care-seeking was the strong belief in observing festivals, which can delay care-seeking.

“*Doctor asked us to take her to Lucknow…it was* Karva Chauth *[festival] that day…we were all fasting so we decided to return home for* Karva Chauth *and take her the next day...”* (sister-in-law)

Men are not involved in discussions of PPH and often are unware of their wives’ experience of it. Women reported feeling shameful to discuss bleeding after childbirth with their husbands since bleeding is considered exclusively women’s domain.

*“I did not tell my husband about the bleeding…I feel shy…it is ‘embarrassing’ to tell men…”* (woman with six children)

One woman mentioned that PPH was too trivial to be discussed with her husband, who stayed far from their village in a city. Husbands were unaware of women’s conditions, and there were discrepancies between husbands and women’s responses about PPH. Interviews with husbands showed their involvement in care-seeking was restricted only to purchasing medicines from private clinics or nearby medical stores. An example of such a discrepancy is the following from an interview in which a woman said,

*“I had asked my husband to get medicines for bleeding and for loose motions.”*

However, her husband said,

“*She had told me to get medicines for hand and leg pain. So I got that. I am not aware of the fact that she had bleeding issue.”*

#### Families’ perceptions of frontline workers

Families did not perceive FLWs to be the first point of contact for most newborn illnesses. As expressed in the quote below, they perceived that FLWs are more active in escorting pregnant women to the institutions for delivery because they are paid for it.

*“ASHA only comes to take pregnant women for delivery, since she gets cash incentive…but because there is no incentive to take newborn child to hospital, so ASHA does not come.”* (mother-in-law)

Therefore, families believed that FLWs do not get cash incentives for taking newborns to the hospital. Families also reported that FLWs are not responsible for newborn care. As a result, families generally do not contact FLWs in case of newborn illness. The findings also show that only three families approached FLWs as the first step of care. When contacted, the FLWs accompanied the mothers to the facilities for the care of their newborns.

#### Transportation

The families believed that newborns were more vulnerable to evil spirits and cold weather, and lack of an enclosed vehicle was described as a barrier to care-seeking for newborn illness. Families associated an ambulance with delivery cases and thus rarely called them for post-partum complications and newborn illnesses.

*“I waited for the night to pass…my mother-in-law said that we will take the child for check up in the morning…as it was very cold outside…there was no enclosed vehicle to take us.”* (woman with three children)

## Discussion

The study was conducted in communities where a maternal and newborn health awareness program was implemented through women organized in SHGs. The study explored the care-seeking behavior among SHG and non-SHG families that faced maternal and newborn illnesses and deaths. Illness recognition is facilitated by prior experiences of self or others with similar illnesses, and severity of symptoms is recognized in terms of frequency and extent of symptoms and change in normal behavior. Older and experienced women are the decision-makers whether care should be home-based or at the community or facility level. Perceptions of cause of illness as medical or supernatural is one of the main factors in decision-making about the type of care provided, whether a traditional healer or other healthcare providers. Other factors influencing care-seeking are timing of the onset of illness and cultural beliefs of post-childbirth confinement. Availability of private providers closer to the communities who are popular, perceived to be good, and accessible at times of emergency facilitates care-seeking. Unavailability of an enclosed vehicle that can protect from cold, perception that FLWs are to facilitate deliveries and not for newborn illness, cultural practices of confinement of the new mother and her newborn to the home, and the cultural consideration about the inappropriateness to discuss issues around childbirth with men are barriers to early care-seeking for newborn and maternal illnesses.

In our study, perceived supernatural causes prompted families to approach traditional healers rather than other health providers. Other studies from UP and Delhi, India, reported that having traditional providers or family members provide care using traditional home remedies was due to perceptions that the quality of care available at local health centers was poor [13, 14]. In contrast, our study showed that not accessing medical health services was due to a lack of faith in the ability of the modern system of medicine to deal with supernatural causes of illness and attributing the outcome of newborn illnesses to destiny.

Studies from UP and Rajasthan, in India, discuss that there are certain ailments, like bulging fontanelle, chest in-drawing, rapid breathing, prematurity, and delayed crying after birth, for which traditional healing was preferred. Only if the traditional medicines do not work, do the families access care from medical practitioners, delaying access to appropriate care [14–16]. Our study confirms previous findings that families preferred home treatment as the first course of action for almost all newborn symptoms, followed by modern treatment if the child did not get better [13].

A cross-sectional study, conducted in the same geographical area showed that maternal and newborn practices like having at least three antenatal check-ups and delivery/complication preparedness, early and exclusive breastfeeding, skin to skin care, and clean cord care were significantly higher among newborns from SHG households compared to non-SHG households (*p* < 0.05) [17]. The present study showed that during pregnancy and early post-partum, both SHG and non-SHG households are rooted in cultural practices, although SHG households sought care from government facilities earlier in the illness event than non-SHG households.

In addition to the existing health intervention through the SHG platforms which is reaching 65,000 SHGs as on date [11], the effects of cultural restrictions around childbirth and its effect on care-seeking in SHG health meeting may strengthen the program. Maternal and newborn illnesses occurring within communities could be discussed in SHG meetings, in order to understand the barriers to care-seeking, find appropriate solutions, facilitate care-seeking, and create awareness. During the SHG meetings, maternal and newborn illnesses and deaths could be discussed as case studies for better understanding of barriers and facilitating factors in appropriate care seeking. Addressing the ways by which cultural norms and rituals become hindrances to appropriate care-seeking is necessary, possibly by ensuring that SHG leaders make frequent home visits after delivery to check on the health of new mothers and their newborns.

The FLWs are key decision-makers for choice of facility and facilitators to reach the facility by arranging ambulance services for illness during pregnancy. FLWs could be invited to SHG forums to discuss case studies and identify barriers and facilitators. SHGs should be made aware that FLWs can be contacted to consult about newborn illness, and if needed, government ambulances can be called to take the newborn to the hospital. The GoI could increase visibility of FLWs and ambulance services for newborn care through communication activities. Further, since FLWs’ participation in women’s group for participatory learning and action (PLA) has shown to be effective in improving newborn survival; therefore, FLWs may be actively involved in the SHG meetings, at least once a week, and apply PLA approaches to improve programs [9].

Furthermore, women with PPH could be treated effectively if the GoI’s policy for women to stay for at least 48 h at the health facility after delivery is strictly implemented [18]. However, understanding beneficiaries’ perspectives regarding their inability to stay for 48 h at facilities and possible ways of addressing this concern through SHGs can be further explored.

## Conclusions

Our study shows that care-seeking is dependent on the perceptions of causes of illness, time of onset of illness, and cultural beliefs and practices around childbirth. Though cultural practices hinder prompt care-seeking for maternal and newborn illness among both SHG and non-SHG families, there are indications that perhaps SHG households are seeking care earlier than non-SHG families, although a large quantitative study is required to test this hypothesis. SHG platforms could be used to discuss myths, misconceptions, and cultural barriers around maternal and newborn illnesses and deaths using their own community experiences. Furthermore, the Government of India could increase awareness of FLWs and ambulance services for newborn care and actively get the FLWs to work with SHG platforms.

## Abbreviations

ASHA: Accredited Social Health Activist
FLW: Frontline health worker
GoI: Government of India
MIS: Management information system
MMR: Maternal mortality ratio
PPH-Post: Partum hemorrhage
RGMVP: Rajiv Gandhi Mahila Vikas Pariyojana
SHG: Self-help groups
UP: Uttar Pradesh

## References

[1] World Health Organization. Analysis and interpretation of the 2015 estimates. In: Trends in maternal mortality 1990 to 2015: estimates by WHO, UNICEF, UNFPA, World Bank Group and the United Nations Population Division. 2015. http://apps.who.int/iris/bitstream/10665/194254/1/9789241565141_eng.pdf?ua=1. Accessed May 2016.

[2] Government of India. Maternal health. Annual report 2014–2015. Ministry of Health and Family Welfare. p. 2015. https://mohfw.gov.in/sites/default/files/5665895455663325.pdf. Accessed 15 June 2016

[3] Herbert HK, Lee ACC, Chandran A, Rudan I, Baqui AH (2012). Care seeking for neonatal illness in low- and middle-income countries: a systematic review. PLoS Med.

[4] Thaddeus S, Nangalia R, Vivio D (2004). Perceptions matter: barriers to treatment of postpartum hemorrhage. J Midwifery Womens Health.

[5] Kumar V, Mohanty S, Kumar A, Misra RP, Santosham M, Awasthi S, et al. Effect of community-based behaviour change management on neonatal mortality in Shivgarh, Uttar Pradesh, India: a cluster-randomised controlled trial. Lancet. 2008;372:1151–62. 10.1016/S0140-6736(08)61483-X 18926277

[6] More NS, Bapat U, Das S, Alcock G, Patil S, Porel M (2012). Community mobilization in Mumbai slums to improve perinatal care and outcomes: a cluster randomized controlled trial. PLoS Med.

[7] Dongre AR, Deshmukh PR, Garg BS (2009). A community based approach to improve health care seeking for newborn danger signs in rural Wardha. India Indian J Pediatr.

[8] Tripathy P, Nair N, Barnett S, Mahapatra R, Borghi J, Rath S (2010). Effect of a participatory intervention with women’s groups on birth outcomes and maternal depression in Jharkhand and Orissa, India: a cluster-randomised controlled trial. Lancet.

[9] Tripathy P, Nair N, Sinha R, Rath S, Gope RK, Rath S (2016). Effect of participatory women’s groups facilitated by Accredited Social Health Activists on birth outcomes in rural eastern India: a cluster-randomised controlled trial. Lancet Glob Health.

[10] Moran AC, Charlet D, Madhavan S, Aruldas K, Donaldson M, Manzi F, et al. Methodology for a mixed methods multi-country study to assess recognition of and response to maternal and newborn illness. Journal of Health, Population and Nutrition (41043–36-S1-S1 in this issue). 10.1186/s41043-017-0119-8 PMC5764055 29297390

[11] RGMVP. Rajiv Gandhi Mahila Vikas Pariyojana: Programs & Impact. 2016. http://rgmvp.org/program-impacts/. Accessed 24 Oct 2016.

[12] Huberman AM, Miles MB, Denzin NK, Lincoln YS (1994). Data management and analysis methods. Handbook of qualitative research. Thousands oak: sage.

[13] De Zoysa I, Bhandari N (1998). Care seeking for illness in young infants in an urban slum in India. Soc Sci Med.

[14] Awasthi S, Verma T, Agrawal M (2006). Danger signs of neonatal illnesses: perceptions of caregivers and health workers in Northern India. Bull World Health Organ.

[15] Mohan P, Iyengar SD, Agarwal K, Martines JC, Sen K (2008). Care-seeking practices in rural Rajasthan: barriers and facilitating factors. J Perinatol.

[16] Awasthi S, Srivastava NM, Pant S (2008). Symptom-specific care-seeking behavior for sick neonates among urban poor in Lucknow, Northern India. J Perinatol.

[17] Aruldas K, Kant A, Hazra A, Mohanan P (2015). Interventions through social platforms for recognition and care seeking of maternal and newborn illnesses in Uttar Pradesh, India.

[18] Government of India. Indian public health standards for community health centres. In: Indian public health standards (IPHS) guidelines for community health centres. Revised. 2012. http://health.bih.nic.in/docs/guidelines/guidelines-community-health-centres.pdf. Accessed 9 Nov 2016.

